# In Search of Commonality: Physiological and Therapeutic Potentials of Sodium-Glucose Transport Protein 2 Inhibitors in Cardiovascular and Chronic Kidney Diseases

**DOI:** 10.7759/cureus.97446

**Published:** 2025-11-21

**Authors:** Khalid Sawalha, Lana Alamat, Aakash Rana, Landon Bruich, Angel Lopez Candales

**Affiliations:** 1 Cardiovascular Disease, University of Arkansas for Medical Sciences, Little Rock, USA; 2 Cardiometabolic Medicine, School of Medicine, University of Missouri-Kansas City, Kansas City, USA; 3 Internal Medicine, Jordan Hospital, Amman, JOR; 4 Medicine, Central Arkansas Veterans Healthcare System, Little Rock, USA; 5 Internal Medicine, University of Arkansas for Medical Sciences, Little Rock, USA; 6 Cardiovascular Medicine Division, School of Medicine, University of Puerto Rico, San Juan, USA

**Keywords:** cardiovascular diseases, chronic kidney diseases, clinical outcomes, outcomes, prognosis, sodium-glucose transport protein 2 inhibitors

## Abstract

Cardiovascular diseases (CVD), including atherosclerotic cardiovascular disease (ASCVD), remain the leading cause of disease burden across the globe, and their prevalence continues to rise outside high-income areas. Furthermore, CVD rates are now showing an upward trend in many locations where rates were previously declining. Despite all the knowledge accumulated about prevention and treatment, many issues remain unaddressed, and this gap between knowledge and prevention continues to widen. The importance of the kidneys in the whole CVD spectrum should be placed in proper perspective, as chronic kidney disease (CKD) is directly linked to the increasing age of the population and is more prevalent among individuals afflicted with obesity, hypertension, and diabetes mellitus - all conditions that influence CVD. Moreover, failure to implement detection strategies and suboptimal prevention strategies for CKD may be contributing to its overall impact on CVD mortality. Within these lines, the use of recent therapeutic interventions, such as sodium-glucose transport protein 2 inhibitors (SGLT2i), may well provide corrective effects on the heart-kidney relationship, particularly in patients with diabetes-related renal disease. Therefore, in this review, we sought to be diligent in identifying which events are responsible for triggering the first "wave" that causes initial destabilization between kidney and cardiac function. Additionally, we aim to provide a comprehensive overview of SGLT2 inhibitors and their pathophysiology in mediating the unquestionable benefits to both the heart and kidneys.

## Introduction and background

According to data from the Global Burden of Disease (GBD) 2019, cardiovascular diseases (CVD), including atherosclerotic cardiovascular disease (ASCVD), remain the leading causes of disease burden across the globe [1]. Prevalence of CVD continues to rise outside high-income areas, while CVD rates are now showing an upward trend in many locations where rates were previously declining [1]. These grim facts indicate that despite all the knowledge we have accumulated, many issues remain unaddressed. In addition, we have been unable to identify which interventions are most effective in lowering the damaging effects of CVD across the entire population [1]. This knowledge gap continues to widen. Those lines uttered by Sir Arthur Conan Doyle through Sherlock Holmes should now make more sense: "When you have eliminated the impossible, whatever remains, however improbable, must be the truth."

One aspect of this main problem may be related to chronic kidney disease (CKD) [2,3]. Approximately 13.4% of the entire world population (between 700 million and one billion individuals worldwide) is afflicted with CKD. Therefore, the importance of the kidneys in the whole CVD spectrum should be placed in proper perspective. Particularly, since CKD is directly linked to the increasing age of the population and is more prevalent among individuals afflicted with obesity, hypertension, and diabetes mellitus [4], all of which have an enormous impact on CVD.

Despite recent advances in identifying atherosclerotic cardiovascular disease (ASCVD), it remains the leading cause of morbidity and mortality worldwide [5-7], including in the United States, where it affects most ethnic groups, a situation that has been attributed to suboptimal implementation of preventive strategies as well as to poor control of modifiable risk factors [5-7]. Therefore, in this review, we sought to be diligent in identifying which events are responsible for triggering the first “wave” that causes initial destabilization between kidney and cardiac function. Additionally, we aim to provide a comprehensive overview of SGLT2 inhibitors (SGLT2i) and their pathophysiology in mediating the unquestionable benefits to both the heart and kidneys.

## Review

Potential contributors to severe CVD

Heart failure (HF) is a growing CVD problem. According to recent estimates, approximately 64 million people are affected worldwide with HF [8]. More disturbing is the fact that HF incidence is expected to continue to rise despite our best efforts [8]. In the United States, age-adjusted HF death rate estimates per capita have continued to increase based on data analyzed from 2009to 2014 [9]. Although divergent trends in mortality have been seen between HF patients with reduced and those with preserved ejection fraction (HFpEF) [10], the prevalence of patients with HF and preserved ejection fraction continues to increase, with alarmingly high rates of non-cardiovascular deaths [11]. No longer should HFpEF be seen as simply left ventricular diastolic dysfunction, but as a group that, in addition to having small and thick left ventricles with four abnormal diastolic filling patterns as their main pathophysiologic abnormality, also has a whole host of different abnormalities. This heterogeneous clinical entity embodies numerous mechanisms and is linked to multiorgan dysfunction, with hypertension and obesity playing a major role. Although we have gained an enormous amount of understanding not only of the causes but also the downstream effects of HFpEF, there is still much to be learned before we can fully comprehend this complex clinical entity.

Among other significant risk contributors to the severity of cardiovascular diseases is peripheral artery disease (PAD), specifically atherosclerotic disease leading to peripheral artery obstruction, which may be silent or present with a variety of symptoms and signs indicative of extremity ischemia [12,13]. The clinical manifestations of arterial insufficiency are due to a lack of blood flow to the musculature relative to its metabolism, which results in pain in the affected muscle groups [13]. The presence of clinical atherosclerosis in one vascular territory, such as carotid artery disease, PAD, or abdominal aortic aneurysm, generally indicates an increased likelihood that it exists elsewhere since the risk factors are generally the same. These shared risk factors might include hypertension, smoking, diabetes, obesity, hyperlipidemia, and non-alcoholic fatty liver disease [12-18].

About cardiac arrhythmias, atrial fibrillation (AF) has been recognized as the most common arrhythmia in the world; it accounts for significant morbidity and mortality [19]. As is the case for both ASCVD and HF, the prevalence of AF continues to increase with the age of the population [20]. Nearly 1.5% of the general population, aged 55-59 years, is afflicted with AF; the incidence rises to 27% in individuals older than 85 years of age [20]. Of all the potential mechanisms currently known to trigger AF, atrial myopathy should be considered extremely important [21]. Atrial myopathy has been characterized by impairments in left atrial structure, function, and electrical conduction. Not only is it critically important in the development of both AF and HF, irrespective of left ventricular ejection fraction (LVEF), but it also leads to a progression of these conditions [22].

Stroke is another CVD contributor to global morbidity and mortality. Based on current estimates, the worldwide stroke burden continues to rise [23-25]. Over 12.2 million new stroke cases are seen each year worldwide [26], with more than 795,000 strokes per year in the United States [27]. Pulmonary hypertension (PH), although not directly seen as a major CVD entity, must be included as a potential factor. First, because of the inordinately high morbidity and mortality associated with the development of elevated pulmonary pressures [28]. Second, and possibly most importantly, because of the whole host of conditions that result in PH [28]. Given this broad spectrum of entities that can cause PH [29], PH-related mortality continues to increase across the five well-recognized World Health Organization (WHO) groups, particularly WHO groups 2-5 [29].

Possible role of CKD

This common condition is critically involved with all the above-listed entities. Aside from infectious illnesses, CKD should be considered one of the greatest threats to mankind. When seen from a proper perspective, half of the patients with CKD stages 4 to 5 have CVD, and up to 50% of all deaths are attributed to CVD events [30-33], including myocardial infarction (MI), stroke, HF, and fatal arrhythmias [34]. In addition, PH is highly prevalent among patients with CKD [35], is commonly encountered in those with ESRD on dialysis, and is associated with increased mortality [36].

Although uremic toxins have always been thought to contribute to the increased CVD morbidity among patients with ESRD [37], two decades after that initial observation, we are still far from fully understanding the global impact of all potential uremic toxins and which mechanisms are critically involved in mediating CVD complications [38].

Currently used criteria for defining and classifying CKD are based on guidelines provided by both the National Kidney Foundation Kidney Disease Outcomes Quality Initiative and the International Kidney Disease Improving Global Outcomes group [39,40]. Based on these recommendations, a fall in kidney function as reflected by reductions in glomerular filtration rate less than 60 mL/min/1.73 m^2^ present for longer than three months, hurts the kidney, including the presence of albuminuria [41-43].

In routine clinical practice, estimated glomerular filtration rate (eGFR) and the presence of albuminuria are indicators of worsening renal function; hence, increased CVD risk [44-46]. Mild reductions in eGFR 60-69 mL/min/1.73 m^2^ have been linked to a higher burden of subclinical atherosclerosis, greater risk of CVD, and CKD progression [47]. Despite recommendations from several guidelines, albumin screening remains low [48-51]. This simple practice must improve, as the presence of albuminuria has been linked to the development of arterial stiffness and, consequently, hypertension, MI, HF, arrhythmia, stroke, and death [52]. Unfortunately, such easily available ways of identifying deteriorating renal function are not put into practice, and their results are used to treat or slow down the progression of such a prevalent disease that has catastrophic implications regarding patient outcomes [51-55].

We agree with Tonelli et al., who concluded their systematic review on CKD by stating that “the risk of developing CVD in patients with CKD surpasses the risk of reaching end-stage kidney disease; therefore, CKD must be considered one of the strongest risk factors for the development of CVD” [54].

Unfortunately, as seen in the most recent 2019 ACC/AHA Guideline on the Primary Prevention of CVD, CKD was only listed as a potential risk-enhancing factor to be used in revising the overall 10-year ASCVD risk estimate of adults with either borderline (5% to <7.5%) or intermediate (≥7.5% to <20%) overall risk [56]. Furthermore, CKD was only mentioned a second time in the entire document to make the obvious assertion that a more intensive approach to controlling hypertension is needed to reduce CVD events [56]. It is important to highlight that this intervention alone has not shown any demonstrable effect in reducing deterioration of renal function [56].

Other factors

Three other entities deserve consideration. First, age is a well-known risk factor common to many conditions; CKD is no exception [57-62]. We currently lack a complete understanding of all potential pathways involved in renal aging and how to differentiate which structural and functional changes distinguish these aging processes from incipient kidney diseases [61]. Second, what role, if any, does gender play in CVD and CKD? Third, what role does race play? In the case of gender, the lack of inclusion of women in prior CVD studies might have interfered with our ability to discriminate if indeed a gender difference exists in CKD and CVD [63-64].

Recent data still urge the need for more women to be equally represented in clinical trials [65]. However, data from the Bogalusa Heart study demonstrated that in both African American and female patients, subclinical atherosclerosis and structural renal damage can be identified early by the presence of albuminuria [66]. More recently, data analyses provided by Carrero et al. and others [67,68] have shown that treatment of kidney failure with dialysis is higher among men, while the prevalence of CKD is now greater among women [68]. It is a fact that in the United States, African Americans are afflicted with high rates of CKD and ESRD when compared to other racial and ethnic groups [69,70]. Similarly, the worst indices of CVD health are also seen among African Americans [71,72]. The occurrence of both CKD and CVD in this ethnic group requires more research. Their presence, however, seems to be a starting point for their pathophysiological links and how to tailor interventions that might be more appropriate to improve overall outcomes.

Cardiorenal syndrome

Cardiorenal syndrome (CRS) is a term that refers to a collection of disorders involving both the heart and kidneys. It encompasses multi-directional pathways between the two organs mediated through low arterial perfusion, venous congestion, and neurohormonal activation. Pathophysiology is complex and includes hemodynamic and neurohormonal changes, but inconsistent findings from recent studies suggest that this is a very heterogeneous disorder. Management for acute decompensated HF remains focused on decongestion and neurohormonal blockade to overcome the intense sodium and fluid avidity of the CRS [72].

A 2004 report from the National Heart, Lung, and Blood Institute defined CRS as a condition in which therapy to relieve congestive symptoms of HF is limited by a decline in renal function as manifested by a reduction in GFR [73,74]. The reduction in GFR was initially thought to result from a reduction in renal blood flow. However, various studies have demonstrated that CRS interactions occur in both directions and a variety of clinical settings.

In 2019, the American Heart Association (AHA) classified CRS into five types: type 1 with acute CRS syndrome, which is characterized by a rapid decline in cardiac function that leads to acute kidney injury. This type of CRS occurs in the setting of active cardiac disease, such as acute decompensated HF, type 2 with chronic CRS, which is characterized by chronic abnormalities in heart function that lead to kidney injury and/or dysfunction. This type of CRS occurs in the setting of chronic heart disease, type 3 is defined as acute reno-cardiac syndrome, which is characterized by acute worsening of kidney function that leads to heart injury and/or dysfunction, type 4 with chronic reno-cardiac syndrome, which is characterized by CKD that leads to heart injury, disease, and/or dysfunction, and type 5 that is associated with secondary CRS, which is characterized by systemic conditions that lead to simultaneous injury and/or dysfunction of the heart and kidney [73,74].

The recent recognition of CRS as an important spectrum of disorders involving both the heart and kidneys [74] serves to point towards the important relation between the two organs. Although CRS occurs in patients afflicted with HF, the imbalance between the two organs may cause acute or chronic changes in the function of the other. Thus, in essence, it magnifies the intricate and close relationship between these two organs [74]. Aside from this close interplay, the kidneys provide a very dynamic, proliferative, and degenerative activation [75] of damaging pathways. Furthermore, in addition to recognition of new molecules, the already well-known interplay of oxidative stress in atherogenesis and inflammation is are pathways that need to be better defined to get to the root cause linking CKD to CVD [76].

Inflammation

There is evidence that chronic inflammatory pathways provide a linking commonality between CKD and CVD. These pathways might join forces and simply initiate and perpetuate mechanisms leading to the detrimental phenotypic expression of both conditions [77, 78]. Notwithstanding, the formation and activation of inflammasomes could be an ideal starting point in both organs. Specifically, the NACHT, leucine-rich repeat (LRR), and pyrin domain (PYD)-containing protein 3 (NLRP3) inflammasome has recently surfaced as a key component [79]. This intracellular sensing protein complex has been shown to play a critical role in innate immunity [80]. Current data suggest that following injury, activation of the NLRP3 inflammasome causes a cascade effect of cytokine production, primarily interleukin (IL)-1β and IL-18 [81]. A well-balanced inflammatory response allows tissue healing with resolution of the initiating triggering injury event; however, excessive NLRP3 activation ultimately may have untoward complications [82,83].

The pro-inflammatory milieu involving the NLRP3 inflammasome, a cytosolic multiprotein complex that activates caspases and promotes the release of IL-1β and IL-18, is found in cells both inside and outside the kidneys and is recognized as a critical pathway in the development and progression of CKD [84]. Furthermore, factors such as uremia, dyslipidemia, metabolic syndrome, intestinal dysbiosis, and malnutrition might, in turn, fuel chronic inflammatory signals in CKD to perpetuate the CVD risk [85].

In the heart, the role of NLRP3 inflammasomes has been studied as a critical path in the development and progression of CVDs over the last decade [86-88]. Our view of the traditional atherosclerotic plaque has significantly evolved from one simply limited to a complex interplay between lipid deposition, peroxidation, and biochemical modifications altering cell migratory patterns and behavior that ultimately lead to arterial wall injury to a more intricate path involving inflammatory changes [89,90].

Even though the field has continued to expand, we have not fully elucidated all involved pathways. It is sufficient to say that aside from NLRP3 inflammasome activation, the participation of other non-immune cells, such as endothelial and cardiac fibroblasts, has emerged as role players in cardiac innate immunity [91]. These interactions have been seen with inflammation in atherosclerosis, ischemia/reperfusion injury, post-MI, and HF [86,91].

Another pathway that requires attention is the thrombospondin family [92]. Data have shown that these large and extracellular matrix glycoproteins, which mediate cell-cell and cell-matrix interactions, are implicated in the development of stroke, cardiac remodeling and fibrosis, atherosclerosis, and aortic aneurysms [93]. A study by Huang et al. was the first to link thrombospondin with CVD among hemodialysis patients [94]. These investigators measured thrombospondin-1 (TSP-1) levels, known to be involved in the regulation of angiogenesis, inflammation, and fibrosis [95-97]. They found that TSP-1 concentrations were significantly higher among patients with ESRD and CVD than among patients with ESRD but without CVD [94]. Specifically, higher TSP-1 levels were independently associated with worse clinical outcomes at the end of three years [94].

Data recently published by Julovi et al. [95] using both a murine model and RNA-sequencing data from explanted human hearts seem to suggest that TSP1 is driving the cellular effects [95]. Consequently, TSP1 might explain mechanisms such as oxidative stress, senescence, and inflammation linking CKD and the development of CVD [95-98].

The introduction of sodium-glucose transport protein 2 inhibitors (SGLT2i) is a promising tool for improving the results of the interaction of CVD and CKD. This novel antihyperglycemic agent class, initially intended to offer better glycemic control to diabetics [99], has revolutionized our approach to treating patients instead with its beneficial effects in protecting the heart and kidneys while improving CKD and CVD outcomes [100,101].

Therefore, to provide at least a basic understanding of the most recent mechanistic benefits currently attributed to SGLT2i, including cardiac and kidney protection as well as overall cardiorenal effects, we have summarized the recent literature in Table 1 [102-163]. Furthermore, we would like to review the currently available and widely used SGLT2i: canagliflozin, which is available in 100 mg and 300 mg tablets; dapagliflozin, available in 5 mg and 10 mg tablets; empagliflozin, available in 10 and 25 mg tablets; ertugliflozin, available in 5 and 15 mg tablets; and the recently approved bexagliflozin, available as a 20 mg oral tablet [164].

**Table 1:** Recognized sodium-glucose transport protein 2 inhibitor-mediated cardiac, renal, and cardiorenal protective mechanisms. IL-6, interleukin 6; EPO, erythropoietin; HbA1c, hemoglobin A1c; NF-κB, nuclear factor κB; ATP, adenosine triphosphate; NHE-1, sodium-hydrogen exchanger 1; TNFR1, tumor necrosis factor receptor 1; MCP-1, monocyte chemoattractant protein 1; RAAS, renin-angiotensin-aldosterone system

Specific protective mechanisms	References
Cardiac protective	
Direct inhibition of sodium/hydrogen (NHE-1) and sodium/calcium transporters in cardiac myocytes alters ion homeostasis, thus reversing cardiac injury, reducing myocardial hypertrophy, fibrosis, and the risk of arrhythmia, while improving remodeling to sustain left ventricular improvement in systolic function.	[101-112]
Reduce myocardial oxygen demand, improve myocardial energetics, and attenuate oxidative stress and inflammation.	[107,108,117-127]
Influence cardiac performance by delaying contracture development during total global ischemia or preserving contractile function during regional ischemia.	[106,125-127]
Improvement in endothelial/vascular function.	[112-115]
An alternative source is β-hydroxybutyrate for cardiac energy.	[112-115]
Reduction in left ventricular mass thus improves diastolic function.	[112-115]
Stimulation of glucagon improves myocardial fuel availability and decreases peripheral vascular resistance.	[112-115]
Reduction in the amount of stressed blood volume.	[112,116]
Renal protective	
Tubuloglomerular feedback and glomerular hemodynamics are affected through the formation of locally vasoactive substances, such as adenosine, resulting from increased sodium chloride delivery to the macula densa. This decreases glomerular perfusion, thus intraglomerular pressure and hyperfiltration, which are central mechanisms for progressive glomerular injury. Reduction of kidney blood flow and increase in vascular resistance in hyperglycemic patients with type 1 diabetes mellitus through afferent arteriolar vasoconstriction. Reduction of glomerular filtration rate through efferent arteriolar dilation.	[128-136]
Reducing tubular workload and mitigating hypoxia in the proximal tubule as a result of hyperglycemia and glomerular hyperfiltration in patients with diabetes through glucose excretion and reductions in urinary albumin excretion.	[137-141]
Promoting diuresis through glucosuria and natriuresis, thereby reducing blood pressure and the risk of heart failure events. The reduction in interstitial fluid in the kidney and decreased proximal tubular energy requirements may alleviate cortical and outer medullary hypoxia.	[142-146]
Anti-inflammatory and antifibrotic effects are achieved through the reduction of levels of NFkB, urinary and serum IL-6, urinary and serum MCP-1, and serum TNFR1. Additionally, SGLT2 inhibitors may reduce serum uric acid levels in patients with type 2 diabetes mellitus.	[147-152]
Cardiorenal effects	
Osmotic diuresis and natriuresis reduce cardiac overload, as SGLT2 inhibitors have shown greater reductions in interstitial fluid compared with other diuretic agents.	[114,153]
Reduction of sympathetic nervous system overdrive.	[154,155]
Improved oxygen delivery through stimulation of renal EPO secretion, therefore increasing hematocrit and hemoglobin.	[156,158]
Reduction in epicardial fat and noxious signals, including leptin and RAAS.	[153,159]
Reducing uric acid, a major promoter of cardiovascular diseases, involves regulating molecular signals associated with inflammatory response, oxidative stress, insulin resistance, endoplasmic reticulum stress, and endothelial dysfunction.	[152,159-161]
Reduction in workload regarding ATP production.	[145,153]
Lowering of HbA1c and body weight.	[153,162]

Despite the undeniable potential clinical benefit, barriers still exist for the use of these agents. For example, in one study, only 32.9% of patients with type 2 diabetes mellitus (T2D) and CKD who were eligible to receive SGLT2i were treated with this drug class [165]. These investigators found that physicians were more glucocentric in their guidance regarding the use of SGLT2i, rather than considering their patients’ tendencies toward overwhelming renal and CVD events [165]. Others have found significant disparities based on racial/ethnic, sex, and socioeconomic differences as barriers to use, limiting prescribing patterns for CVD prevention [166]. A similar disparity related to sex and race was also observed in prescriptions for SGLT2i among likely eligible patients within the Veterans Affairs (VA) Health Care System [166,167,168].

Costs can, of course, present a barrier to the use of these agents; however, clinical practice patterns and providers’ attitudes must be modified through education and the demonstration of beneficial results to improve prescribing of SGLT2i [164]. This unfortunate trend must also be reversed among cardiologists, who should be aware of the recent results from improved cardiovascular outcome trials [169]. The potential risks of euglycemic diabetic ketoacidosis and genitourinary infection must be kept in mind, particularly in patients at higher risk [170]. Finally, we would like to emphasize the well-established benefits of SGLT2i for cardiovascular outcomes, including reductions in hospitalization rates, stroke, kidney failure, myocardial infarction, heart failure, and mortality [170-171].

## Conclusions

In summary, we must strive to close gaps in knowledge and undertake new avenues of investigation that will eventually allow us to better understand the common thread that keeps the heart and kidneys working in proper harmony. We need to establish if, and how, the kidneys are involved in the initial stages that alter atherogenesis and perpetuate metabolic abnormalities. Examining inflammatory pathways may provide alternative strategic checkpoints to introduce reliable therapeutic strategies. Our current understanding of these molecular pathways needs to be expanded and refined to guide us toward improved therapies. Healthy kidneys are essential for maintaining the health status of CVD patients and improving overall outcomes. Even if failing kidney function is not the sole contributor to poor CVD outcomes, it seems to be critically important, and we must continue searching for the best interventions to prevent unnecessary complications. 
